# Thermal and Mechanical Studies of Perlite Concrete Casing for Chimneys in Residential Buildings

**DOI:** 10.3390/ma14082011

**Published:** 2021-04-16

**Authors:** Krzysztof Drozdzol

**Affiliations:** Faculty of Civil Engineering and Architecture, Opole University of Technology, Katowicka 48 Street, 45-758 Opole, Poland; k.drozdzol@po.edu.pl; Tel.: +48-77-449-8581

**Keywords:** chimney, perlite concrete, fire safety, heat recovery

## Abstract

Chimneys are structures designed to convey exhaust gases from heating devices to the outside of buildings. The materials from which they are made have a great impact on their fire safety, as well as on the safety of the whole building. As current trends in the construction industry are moving towards improving the environmental impact and fire safety, changes to building materials are constantly being introduced. This also applies to the development of chimney technology, as there is still a recognised need for new solutions when it comes to materials used in the production of chimney systems. This article presents the findings of tests carried out on a chimney made from innovative perlite concrete blocks. Four different perlite concrete blocks that differed in bulk densities were analysed. The obtained results were then compared with widely used leca (lightweight expanded clay aggregate) concrete blocks. The test results confirmed high insulation properties of the perlite concrete block, from which the innovative chimney casing was made. The fire safety level was maintained even in high temperatures that occur during soot fire (1000 °C). These properties were retained despite there being no additional insulation of the flue duct. Even though the thermal load decreased the compressive strength of the chimney blocks, they still displayed sufficient average strength of 4.03 MPa. Additionally, the test results confirmed the possibility of recovering heat from the chimney with the efficiency of 23–30%, which constitutes a considerable increase compared to chimneys made from leca concrete blocks.

## 1. Introduction

Chimneys are architectural structures designed to convey exhaust gases from heating devices to the outside of buildings. The materials from which they are made have a great impact on the fire safety of those structures, and of the whole building. The side effect of fuel combustion (especially combustion of solid fuels) is the formation of sediments and soot on the internal walls of the flue. When it comes to solid fuels, the exhaust gases themselves are characterised by high temperatures, often reaching 600 °C. In the event of soot combustion, the temperature inside the chimney reaches over 1000 °C. A considerable part of fires is started due to heat radiation from the chimney onto flammable structural elements of the building. This creates significant threat to the residents of buildings heated by fuel-based equipment. Hence, it is vital that the chimney has the highest possible level of insulation. Ecology is just as important as fire safety. The sole purpose of a traditional chimney is to convey smoke into the atmosphere. These chimneys are usually made of brick, insulated steel pipes, and leca (lightweight expanded clay aggregate) concrete blocks with a ceramic duct insulated with wool. Currently, multi-layered chimneys, where the air layer plays a vital part, are becoming increasingly common. The role of the air layer is to feed air into the combustion chamber. This air receives heat from the flue duct, which decreases the temperature of the casing. Furthermore, when fed into the heating appliance, the heated air increases its efficiency. However, the vast majority of scientific literature is focused on chimneys without air layers.

One of the most commonly used chimneys is the double-walled steel chimney, which was thoroughly tested by Leppanen et al. [1,2,3] and Neri et al. [4,5,6,7,8]. These researchers concentrated their analyses on the temperatures at chimney–roof penetrations in the presence of a steel chimney [1,2,3,4,5,6,7,8,9]. Steel chimneys do not allow for heat recovery from exhaust gases, and they are characterised by a lower level of fire safety than chimneys with air layers. Drozdzol [10] analysed the safety of steel chimneys with air layers. His focus was on temperature distribution in different layers of the chimney and on heat radiation from its casing.

Brick chimneys have also been analysed for fire safety. Peacock [11] determined temperature distributions on the external surface of a brick chimney with a ceramic liner during soot fire. Drozdzol [12,13] presented an experimental analysis of fire safety and the efficiency of energy recovery from chimneys made of isostatic ceramics. The casings of the tested chimney systems were made from leca concrete blocks. An evaluation of the safety of a liner made of a composite material was presented by Kererekes [14]. In her analysis, she also emphasised the liner’s fire resistance. The solution presented herein may be helpful in renovating brick flue liners.

Silcock and Shields [15] focused on a different aspect of brick chimneys. They described the validation of processes pertaining to temperature and flow that were computationally and experimentally determined. They tested two chimneys, 150 mm and 200 mm in diameter, with ceramic liners in brick casings. The third solution they tested was a flue liner, 150 mm in diameter, in brick casing, where the space between the casing and the liner was filled with a mixture of perlite and cement. The thermal conductivity of the cement/perlite backfill matrix was taken as 0.18 W/mn·K and the thermal conductivities of the clay casing and brickwork were taken as 0.50 and 0.85 W/m·K. The data concerning thermal conductivity are particularly important from the point of view of this article. Although the literature abounds in studies concerning perlite concrete, research involving this material focuses on its use in wall and roof construction and insulation rather than chimney blocks [16,17,18]. According to the findings of Wang et al. [19], who studied the thermal conductivity and strength of concrete after adding aerogel with perlite, concrete with this addition has better thermal parameters. The expansion of perlite creates dust, which constitutes waste. Rozycka and Pichor [20] proved that adding this dust instead of quartz sand to a mix improves the insulation properties of the product. Nonetheless, the authors emphasised that if the amount of the added dust exceeds 15%, the strength of the concrete may decrease. Rashad [21] published an overview study in which he described the effects of adding perlite instead of aggregate to various concrete products. The above publications confirm the viability of using perlite in the production of chimneys in cases where insulation plays a key role in ensuring the safety of the residents and where heat recovery is desirable.

Thus far, studies have depicted analyses concerning the effect of high temperatures on the physical properties of lightweight concrete [22,23], including lightweight concrete with the addition of perlite [24,25,26,27]. However, these publications do not address issues pertaining to chimney blocks in that respect. The methodology presented and used in those studies cannot be implemented in analyses concerning the chimney’s resilience to soot fire due to the fact that the described tests were conducted on cylindrical and cubic reference samples. Moreover, the samples were subjected to high temperatures in furnaces, where temperature distribution is even. During soot fires in chimneys, high temperatures are only present inside the chimney block. Due to the concentration of stresses in the apertures, as well as second order effects in slim and irregular walls of chimney block elements, these results need to be differentiated.

Maraveas et al. [28,29] demonstrated that there are correlations between the Eurocode mathematical expressions and lightweight concrete properties. They can be used to assess the impact of thermal properties, dependent on temperature, on the mechanical properties of lightweight concrete. The EN1992-1-2 [30] standard does not address the issue of chimney hollow blocks. The products used in the chimney technology are distinctive and should not feature the approach used in the designing of other structures due to fire safety (poles, walls, beams, slabs, etc.). This derives from the geometry of walls which are thin and irregularly shaped as well as from the thermal requirements that must be met.

Another aspect of scientific research is the analysis of chimneys aimed at improving the efficiency of heating appliances, e.g., through heat recovery, which has a positive effect on the environment. Such analyses concerning steel combustion air flues used in low temperature gas appliances were described by Czerski et al. [31,32].

Chimneys with leca concrete casings are insulated with mineral wool whose smouldering decreases the building’s fire safety [4]. Since the development of the construction industry involves increasing both energy savings and fire safety, there is a need for new materials with better insulation properties than leca concrete. It would be advantageous to find lighter materials to use for the casing, which would facilitate transport and reduce its costs.

This article presents findings that determine the properties of chimneys with a ceramic duct surrounded by innovative perlite concrete casing, which has not been used in chimney technology before. Special attention has been paid to the effects of perlite concrete block casing and increased thermal insulation on the efficiency of energy recovery and fire safety. The study also includes an analysis of the blocks’ compressive strength before and after soot fire simulation. The purpose of the study was to find a material with optimised parameters of thermal insulation which would prevent the temperature on the casing from exceeding 100 °C during soot fire and guarantee compressive strength greater than the weight of 100 m high chimney column, i.e., 1.14 MPa. This study had never been done by other researchers. Moreover, the above-mentioned analyses take into consideration both fire safety and the ability to recover heat from the chimney’s air space. The findings of the study are compared with the traditional chimney made of leca concrete blocks insulated with mineral wool.

## 2. Material Characteristics

**Figure 1:**
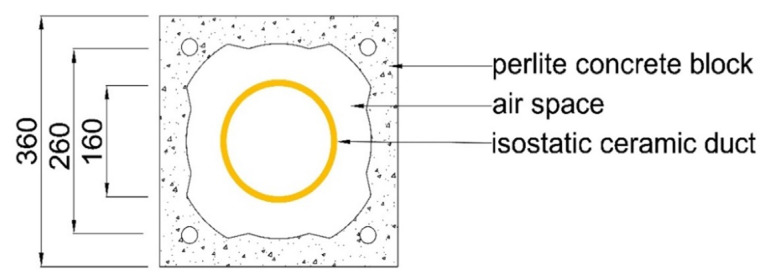
Cross-section of the layers of the analysed chimney (dimensions in mm).

The subject of the study presented herein was a chimney system comprised of three layers (Figure 1): (i) isostatic ceramic flue duct; (ii) air space; and (iii) perlite concrete block.

The main study concerns the innovative perlite concrete block, and the findings are compared with the parameters of widely used leca concrete blocks. Although chimneys made of leca concrete blocks are characterised by sufficient resistance to soot fire, they must be coupled with additional layers of insulation. They are also characterised by relatively low insulation when it comes to heat recovery [13]. Their declared distance from flammable materials is 20–100 mm, and the temperature classes are T400 and T600 depending on the manufacturer [33,34,35]. The bulk density of leca concrete blocks falls within the range of 1042 to 1200 kg/m^3^, and their compressive strength varies from 3–3.5 MPa [33,34,35]. According to the manufacturer, the height of the chimney cannot exceed 20–25 m [33,34]. Expanded clay is an aggregate responsible for the load-bearing capacity and insulating power of leca concrete blocks. Its bulk density is 500–1500 kg/m^3^, its thermal conductivity coefficient λ starts at 0.09 W/m·K, and its strength ranges from 0.7 to 10 MPa [36].

The blocks which constitute the casing of the analysed chimney were made from a concrete mix in which perlite was the main insulant. No studies of this type of chimney casing had ever been presented in any publications before. This is why the first parameter that we determined was the thermal conductivity, which is characteristic of a perlite aggregate in its loose unsettled state. In this case, it was 0.057 W/m·K. The characteristics of the physical properties of the aggregate (perlite) prepared by the supplier are presented in Table 1.

As a result of the initial analysis, four perlite-based concrete mixes (no. 3–6) were selected for further detailed analysis. The samples of perlite concrete blocks were made from concrete mixes composed of: (i) 240 kg CEM 42.5R cement (Cementownia Warta S.A., Trebaczew, Poland) characterised by faster setting time and high early strength (using this type of cement minimises the risk of shrinkage cracks, which are common in perlite concretes); (ii) 330 kg of unwashed sand with fraction of 0–2 mm. This fraction is used due to perlite’s grain size (approx. 1 mm) and had been determined as a result of optimising aggregates to obtain proper compaction; (iii) 135–156 litres water; (iv) perlite added in the range of 1–1.2 m^3^, characterised in Table 1; and (v) completely formaldehyde-free superplasticiser based on non-sulphonic acrylic polymers. Thanks to this admixture, the mix retains its workability even at high temperatures, and obtains high water resistance and mechanical strength parameters after hardening. As a result, blocks with bulk densities ranging 943–1294 kg/m^3^ were obtained. The specific amount of perlite as well as the types and specific amounts of chemical admixtures are not presented herein due to the protection of this data by the manufacturer (trade secret). The different densities of the perlite were obtained by using different grain sizes, as well as different operation durations and frequencies of the vibration press. The mixing was done in Sicoma 1125/750 (Perugia, Italy) planetary mixer, and the casting and compacting was done with the use of an MFS TYTAN 3 vibration press (ZPUH Road Sp. z.o.o.,Włoszczowa, Poland) for concrete prefabrication.

The blocks produced according to the aforementioned method underwent curing in a maturing facility. Thanks to the fact that the curing process took place in a controlled environment, the ambient conditions, i.e., temperature of 35–45 °C and humidity exceeding 95%, could be properly maintained. After 30 days of maturing, the samples were analysed.

### Testing of Perlite and Leca Concrete

**Figure 2:**
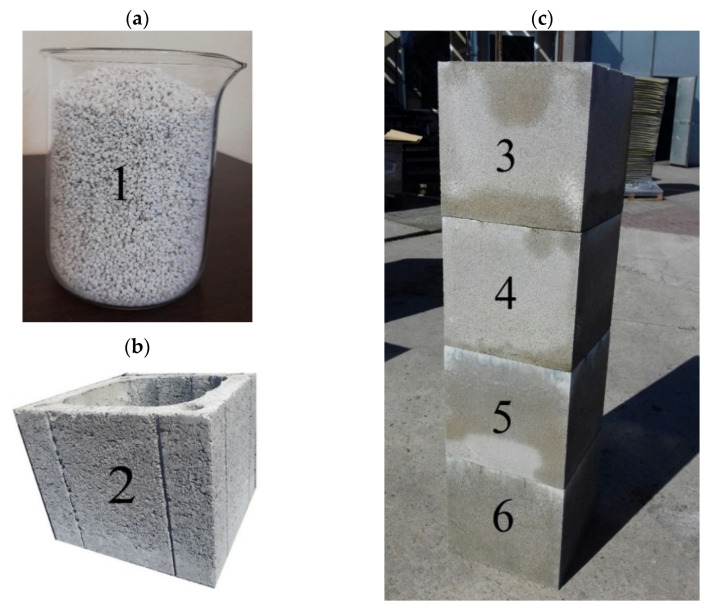
Photos of: (**a**) perlite (1), (**b**) leca concrete block (2) and (**c**) perlite concrete blocks (3–6) with different bulk densities.

After determining the perlite’s thermal conductivity in loose state (sample no. 1, Figure 2a), the thermal conductivity coefficient for leca concrete blocks (sample 2, Figure 2b), which are commonly used in chimney technology, was determined. The leca concrete selected for comparative analysis had a bulk density similar to the average bulk density of the applied perlite concrete. Moreover, it is the most commonly used material on the market. The available information about the thermal conductivity of this type of chimney allows for the comparison of its insulation properties with the innovative chimney which is the subject of this study. The next stage of the analysis involved the determination of thermal conductivity coefficients of the samples removed from perlite concrete blocks (Figure 2c) with different bulk densities of the concrete (no. 2–6). This analysis was conducted in the laboratory of the Department of Materials’ Physics, Faculty of Civil Engineering and Architecture, Opole University of Technology.

**Figure 3:**
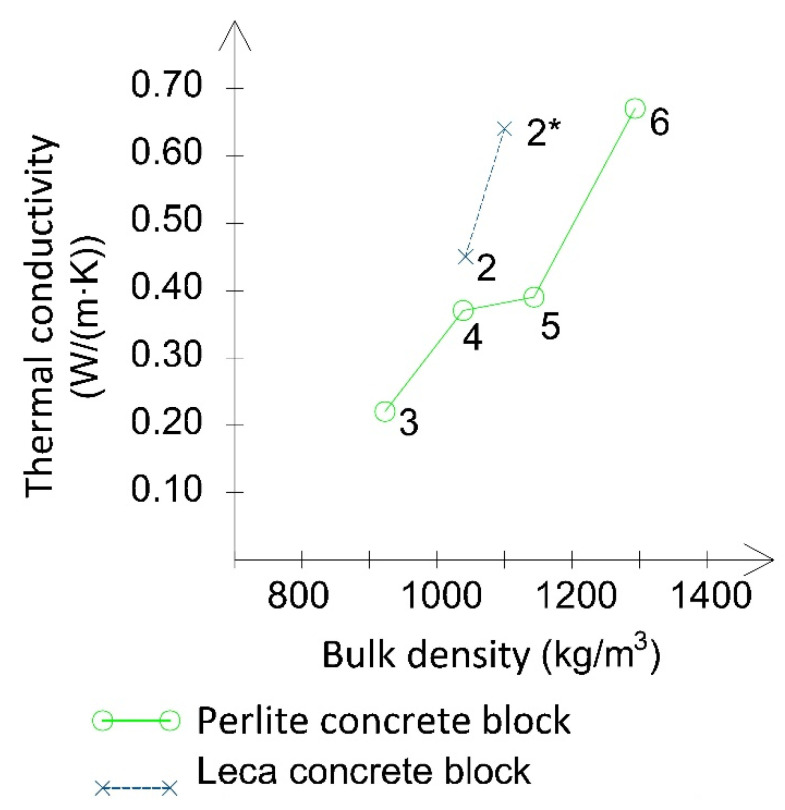
Dependency of the thermal conductivity coefficient on the bulk density of perlite and leca concrete blocks; 2* value determined based on [33,34].

In order to determine the thermal conductivity coefficient, 100 mm × 100 mm samples from the chimney blocks (expanded and perlite concrete) were removed and their temperatures were measured on two sides. The measurements were taken in a steady state in a custom-made test chamber (Opole University of Technology, Opole, Poland). The temperature differences measured during the tests were marked as Δ*T*_1_ and Δ*T*_2_. The average temperatures in the samples and the thermal conductivity coefficients of the tested materials calculated on their basis are presented in Table 2. Additionally, the dependencies between the bulk density and thermal conductivity in perlite concrete samples are illustrated in Figure 3, which shows that the values of thermal conductivity increase with the products’ bulk density. The thermal conductivity coefficient of leca concrete chimney blocks (Table 2, Figure 3) was determined based on the analysis and the data provided by their manufacturers [33,34]. Figure 3 shows that the curve indicating the dependency of bulk density on thermal conductivity is close to linear. The disclosed data indicates that at the same bulk density, the thermal conductivity of the product made from leca concrete is higher than that of perlite concrete by 0.07 W/m·K. This confirms that the insulation parameters of leca concrete are 18% worse than those of perlite concrete. The samples of perlite concrete with different densities were also tested for compressive strength (Table 2) and the results indicate a considerable increase in strength along with an increase of the product’s bulk density. Moreover, it is evident that the compressive strength of leca concrete block samples is higher than the compressive strength of perlite concrete samples at a comparable bulk density.

The strength of the product was tested by compressing corners which had been cut out from the blocks in line with standard EN 12446:2003 [37]. The properties presented by the manufacturers of expanded concrete chimneys are determined based on the same methodology, making it possible for the results to be compared.

The next layer of the chimney was an isostatic ceramic duct which constituted the flue lining. The lining was made from ceramic granulate with the use of isostatic pressing (pressure of approx. 450–500 bars). The bulk density of the granulate was 121.3 g/dm^3^. The granulate consisted of grains of the following sizes: 0.5 mm (20% of the granulate); 0.2 mm (17.6% of the granulate); 0.1 mm (42.1% of the granulate); 0.063 mm (26.2% of the granulate) and less than 0.063 mm (13.9% of the granulate). The product was characterised by low water absorption and low permeability, and was resistant to soot fire. The duct was characterised by conductivity of 0.5 W/mK [15].

When analysing the obtained results of the block’s thermal conductivity, it was noted that the perlite concrete samples were characterised by a 20% lower conductivity than the samples containing expanded clay. This lends support to the production of blocks from this type of material. The measurement findings also indicated that the thermal conductivity of the samples decreased significantly along with a decrease of the block’s bulk density (Figure 3).

## 3. Description of the Analysed Chimney

The exhaust duct of the chimney was made from isostatic ceramics and had the diameter of 160 mm. Its air space was 25 mm in its narrowest point. The external dimensions of the block were 360 × 360 mm (Figure 1). The height of the analysed chimney was 4.10 m.

Due to the fact that chimney blocks characterised by a lower bulk density have a significantly lower compressive strength (Table 2), the block selected for further analysis had a bulk density of 1039 kg/m^3^ (sample no. 4, Table 2), and thermal conductivity of 0.37 W/(m·K). The decision to choose this block was made based on its optimal compressive strength to thermal conductivity ratio.

The flue duct of the chimney was not insulated with mineral wool. This is a significant difference compared to currently produced leca concrete chimneys that contain mineral wool insulation. The resignation from the insulation layer allows a more detailed evaluation of the insulation properties of perlite concrete casings. If the experimental tests confirm that the remaining parameters of the chimney are maintained despite resignation from insulation, future chimneys may not contain mineral wool as insulation, which would decrease production costs. Moreover, wool insulation is prone to smouldering in high temperatures, which further increases the temperatures on chimney casing, thus increasing fire hazard [4].

**Figure 4:**
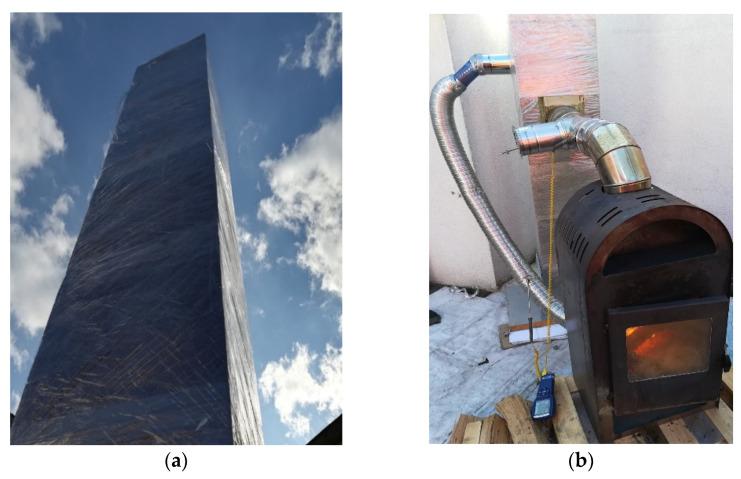
Test photos: (**a**) view of sealed chimney over the roof and (**b**) heating appliance after installation.

The entire length of the chimney was secured (sealed) with foil for the duration of the tests (Figure 4a). Since literature does not contain any guidelines for this type of tests, the applied methodology had been developed for the purposes of the experiment described herein. The chimney was insulated in order to eliminate all possible leaks that could have appeared during installation. Despite utmost care, sometimes small leaks between different layers are formed during installation, as described by Lichtenegger et. al. [38,39]. Sealing the chimney with foil decreased the probability of the air leaking through any cracks that may have appeared at that stage.

## 4. Methodology of Conducted Tests

### 4.1. Heat Recovery from Air Space

#### 4.1.1. General Notes

**Figure 5:**
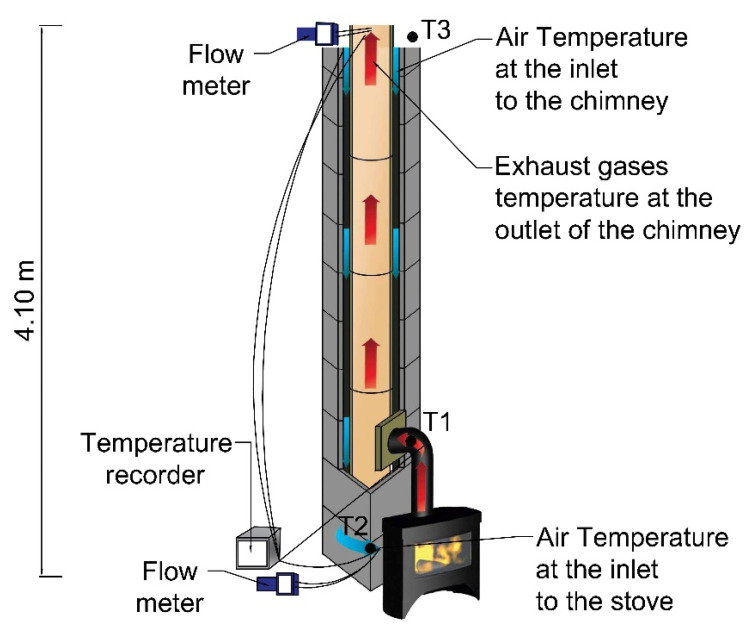
Test stand (blue and red arrows indicate air and exhaust, respectively).

The test stand used for experimental testing is shown in Figure 5. In the analysed system, the air is fed into the combustion chamber in counter current. As it flows through, it is heated by the exhaust. If the air is significantly heated, there is a risk that the flow in the air space is reversed and the system becomes co-current. This risk is increased by the resignation from mineral wool in the chimney. If the amount of the air fed into the combustion chamber is insufficient, the chimney draught is reduced. This results in incomplete fuel combustion and a build-up of CO in the combustion chamber. In turn, this may lead to the release of highly toxic carbon monoxide into the building.

The appliance used for the experimental testing was a stove prototype with a closed combustion chamber (Figure 4b). The stove drew air directly from outside, was equipped with a chimney cowl and a double afterburner system. The stove’s power was estimated at 8 kW, and its efficiency was at 85%. The fuel used for testing was firewood–birch with a moisture content of <20%, which had been confirmed with the use of a meter before the tests began.

The experimental tests made it possible to conclude whether resigning from thermal insulation of a flue duct would affects the functioning of the chimney. Similar ceramic-and-concrete systems have been already studied. However, the flue was additionally insulated with mineral wool, and the block was made of leca concrete [13]. The tests of leca concrete chimneys described in literature allowed the evaluation of their fire safety and the conclusion that the average heat recovery from these chimneys was at 5%.

#### 4.1.2. Measuring Equipment

The tests involved measuring the temperatures of the air and the exhaust, as well as the temperature of the chimney casing. The temperature of the air in the air duct and the temperature of the exhaust were measured with the use of a multi-channel temperature recorder produced by Czach (Czach-Pomiar Sp. z o.o., Katowice, Poland with Type K Class One thermocouples. The thermocouples were distributed as follows: T1–exhaust temperature at the inlet to the chimney; T2–air temperature at the inlet to the stove; and T3–thermocouple measuring the temperature of the atmospheric air. The measuring error for the temperatures was ±1 °C. The measurements were also double-checked with the use of thermocouples connected to TSI 9555-P meter (TSI Incorporated, Shoreview, MN, USA). The velocity of the airflow at the inlet to the stove and the velocity of the exhaust at the outlet of the chimney was measured with flow meter TSI-9555P.

#### 4.1.3. Course of Tests

After building the stand and sealing it with foil (Figure 4), the main testing began, and was divided into three stages:

(Stage 1) Kindling—at this stage, special attention was paid to the ease of fire initiation in the stove. Another important aspect of the observation was obtaining the appropriate chimney draught and air intake to the stove. In order to be sure that the air for combustion was provided from the air duct rather than leaks, the provided air was coloured. Thanks to transparent control points, it was possible to observe the flow of the coloured air and exhaust.

(Stage 2) Conditioning—after kindling, for over 2 h the exhaust temperature was maintained at over 150 °C at the outlet of the chimney. This was done in order to warm up the system and to remove moisture from the chimney to stabilise testing conditions.

(Stage 3) Main testing—these analyses lasted for over 4 h. During this time, the temperature of the exhaust at the outlet was maintained at 150–285 °C and the readings were taken every 15 min. Figure 4 and Figure 5 show the test stand during testing. The temperature of external air was 12 °C ± 2 °C, the atmospheric pressure was 987 hPa, and the wind flow was less than 3 m/s.

### 4.2. Fire Safety Evaluation

The fire safety was studied on a 2.6 m high model, in line with standard EN 13063-1:2005+A1:2007 [40], according to which testing ought to be done on at least 2 m long sections. The temperatures at the inlet to the chimney, on its casing and on flammable materials were controlled during the study. Similar to the previous tests, the study was done on a chimney consisting of a flue duct, an air layer and perlite concrete casing (without additional insulation layers).

#### 4.2.1. Test Stand

**Figure 6:**
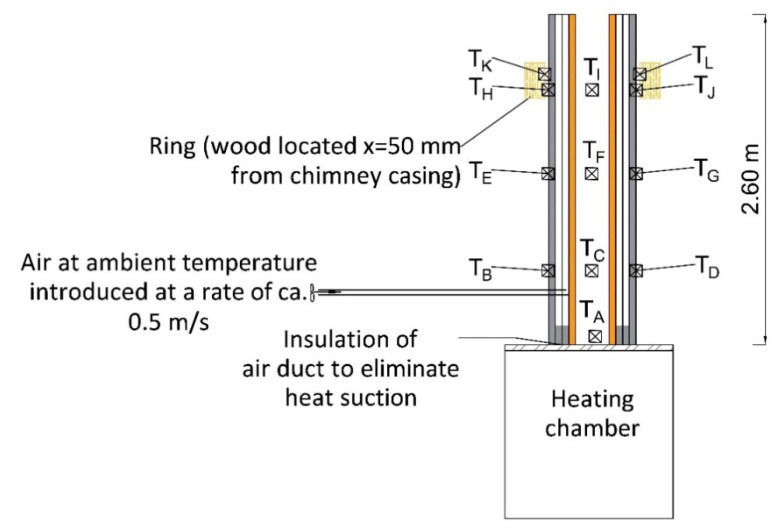
Diagram of the stand used for chimney fire testing.

**Figure 7:**
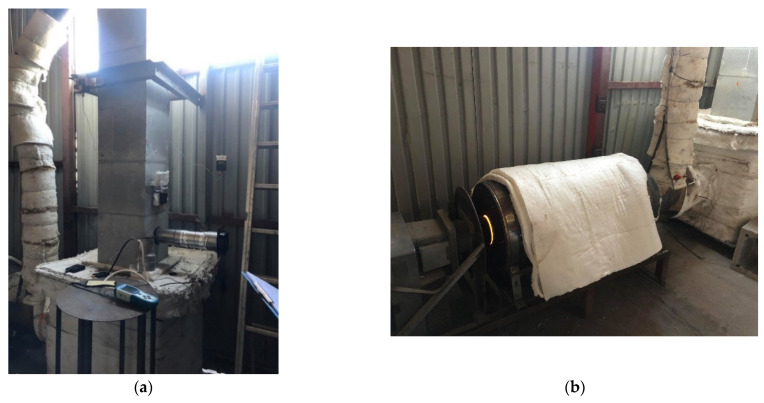
Stand used for fire testing: (**a**) tested chimney on the testing chamber and (**b**) hot exhaust generator.

The test stand (Figure 6) consisted of an oil-powered hot gas generator, which fed hot gases to a chamber that introduced the stream of hot exhaust gases vertically to the chimney. There were flammable wooden materials 50 mm away from the chimney, at a height of 1.80 m (Figure 6 and Figure 7).

Temperature sensors were distributed as follows (Figure 6): (i) at the inlet to the chimney (T_A_); on the circumference of a wooden ring (T_B_–T_J_), and (iii) on the surface of the blocks (T_K_–T_L_) at the heights of 0.55 m, 1.10 m, and 1.80 m respectively. The temperature sensor on the surface of the blocks was additionally insulated on the outside with mineral wool, which limited heat radiation and provided less favourable conditions.

#### 4.2.2. Course of Tests

The hot exhaust was gradually fed into the chimney for 30 min until obtaining a temperature of 700 °C (testing temperature during simulation of the operation conditions is 100 °C higher than the temperature determined by the manufacturer–T600), as recommended by standard EN 13063–1:2005+A1:2007 [40]. Temperature recording began after the temperature reached 700 °C. During testing, the rate of the air fed into the air space was ~0.5 m/s (a value similar to the lowest rate recorded during tests with the use of a stove with a closed combustion chamber). The temperatures were recorded every 5 min and read every 30 min. The operating conditions were simulated for 210 min. At the beginning of the experiment, the ambient temperature in the laboratory room was 12 °C. Soot fire was simulated analogously, by feeding exhaust at 1000 °C into the chimney for a duration of 30 min, with the measured values read every 10 min.

### 4.3. Compressive Strength Test

**Figure 8:**
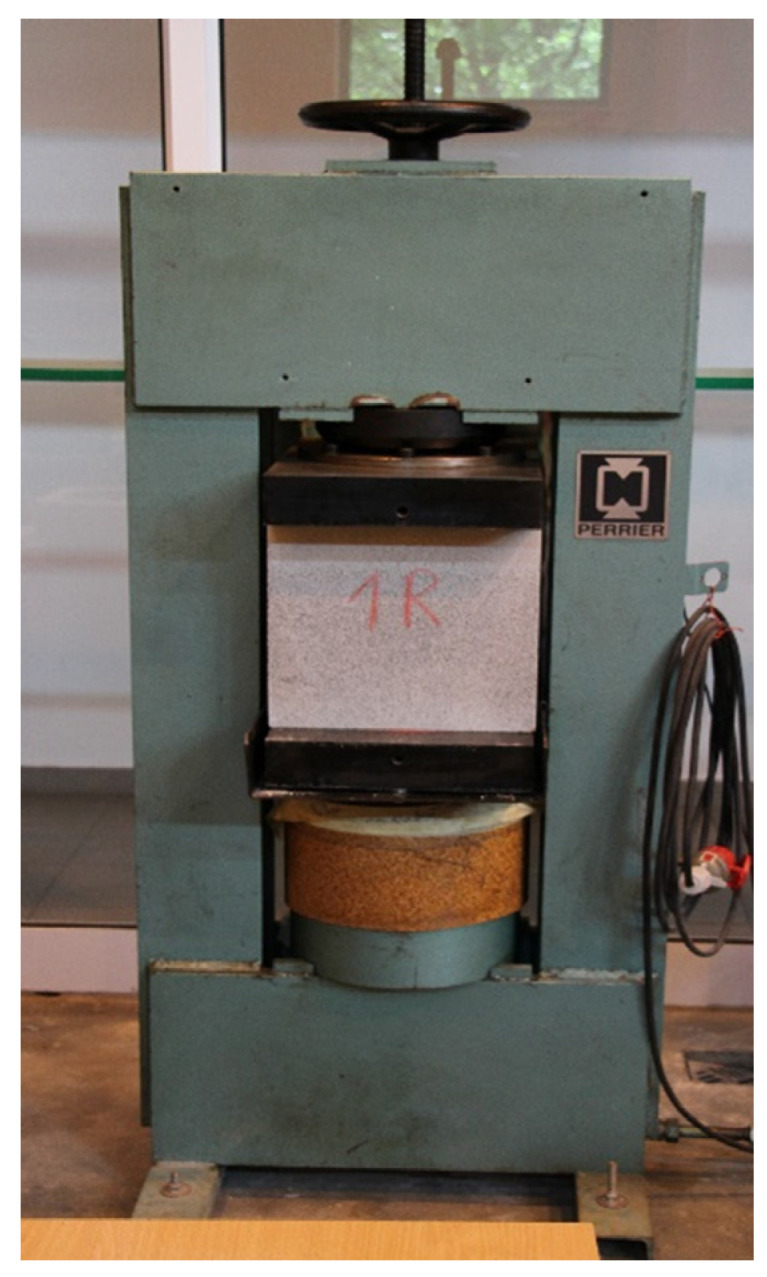
Course of strength testing–compression of a block in a hydraulic press.

Apart from a high level of insulation, a chimney should also have a proper load-bearing capacity. This is why fire testing was followed by tests of the compressive strength (Figure 8) of perlite concrete blocks (based on mix no. 4, Table 2). A chimney should be able to bear its own load even if its structure is weakened due to fire. The tests were carried out on perlite concrete blocks which had not undergone fire testing, and on blocks subjected to thermal testing described in the previous part of this article. During thermal testing, the blocks were subjected to high temperatures, i.e., 700 °C in a simulation of operating conditions, and 1000 °C in a simulation of soot fire conditions.

Information on the strength of brand-new blocks can be used as reference values. Testing strength after applying fire load to the structure made it possible to conclude whether a block would continue to carry loads safely after a fire. The tests involved analysing 6 blocks from each group (before and after thermal testing). The first set of trials was done on those blocks which had not been subjected to thermal testing. They were treated as reference blocks and marked as 1R–6R. The remaining blocks had been under fire load during thermal testing and they were marked as 1F–6F. PERRIER hydraulic machine with a pressing strength of 2000 kN, equipped with a road transformer converter Ptx 200 made by Peltron, and a precision pressure converter type P-30 made by WIKA. Measurements and data acquisition have been performed by the MGC plus system by Hottinger Baldwin Messtechnik (Poznań, Poland) equipped with an amplification module ML801 B. The load was applied at a speed of 1 kN/s.

## 5. Results and Discussion

### 5.1. Heat Recovery

**Figure 9:**
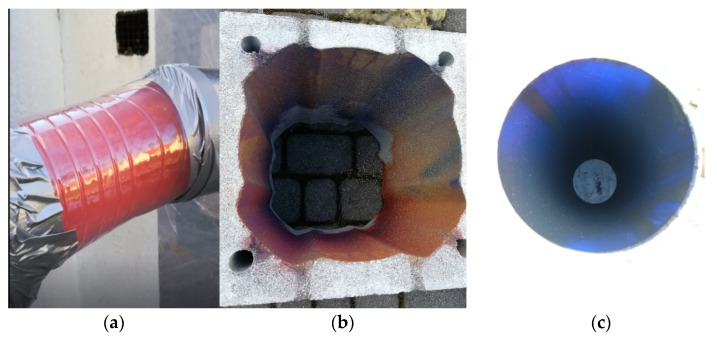
View of (**a**) transparent control point at the inlet of the air to the stove, (**b**) coloured structure of the air duct (red) and (**c**) coloured flue (blue).

This part of the tests mainly involved observation of the performance of the air layer while air was fed into the stove in order to ventilate the combustion chamber. The second aspect of this part of the analysis was the evaluation and determination of the efficiency of heat recovery from chimney loss (removed with exhaust). No problems were observed with obtaining the appropriate chimney draught during kindling. The smoke was properly discharged, and subsequent stages of combustion were passed without any problems. It was concluded that the system worked correctly, the air was fed into the stove through the air duct and conveyed into the atmosphere through the ceramic flue. The tests were carried out with the combustion chamber closed and open. Below are photographs of the test (Figure 9), including screen shots from a video recording, as well as photographs taken after the study, which show coloured elements. This confirms that the gases were flowing through the flue duct. Note the uniform colouration of the chimney elements, which confirms flow through the whole cross-section of the channels.

The temperatures of the air fed into the stove and the discharged exhaust were measured (Table 3). Additionally, the temperature of the chimney casing was measured along its entire length. The highest recorded temperature of the casing was 40 °C, which confirms the high level of the chimney’s operational safety. This temperature was recorded above the elbow connecting the flue to the heating appliance. At the remaining measuring points, the temperature values were up to 12 °C lower.

The flow of the exhaust and air was measured after two hours of testing. The flow measurements were taken when the inlet to the stove was closed and the exhaust temperature at the inlet reached 173 °C. At that time, the flow rate in the flue duct was 0.27 m/s, which means that 0.012 m^3^ of exhaust was flowing through the flue per hour. At the same time, the flow of air through the air space was measured and found to be 0.45 m/s, which means that 0.012 m^3^ of air was flowing through the air space per hour. The same measurements were repeated with an open damper at the inlet to the stove. At that time, the flow rate in the flue duct increased to 0.98 m/s, which means that 0.043 m^3^ of exhaust was flowing through the flue per hour. The flow of air through the air space was 1.60 m/s, which means that 0.044 m^3^ of air was flowing through the air space per hour. After opening the inlet, the temperature increased to 215 °C. The measurements confirm normal flow of the discharged exhaust. At the same time, the amount of air provided to the combustion chamber was sufficient for the purposes of fuel combustion. These results justify the possibility of heat recovery and further analysis of the efficiency of the recovered heat.

Based on the obtained results, the thermal efficiency of the chimney *η_t_*, was determined with the use of the following Equation (1):

(1) $$\eta_{t}=\frac{T_{12}-T_{11}}{T_{21}-T_{11}}\;$$

$T_{12}$–air temperature at outlet, °C,
$T_{11}$–air temperature at inlet (ambient), °C,
$T_{21}$–exhaust temperature at inlet, °C.

**Figure 10:**
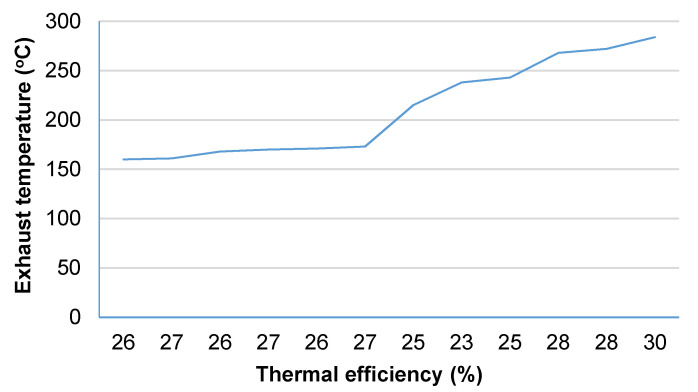
Thermal efficiency of a chimney made of perlite concrete blocks.

Figure 10 shows the efficiency of the chimney (treated as a heat exchanger) determined during the experiment. The obtained efficiency ranged between 23% and 30%. The efficiency of heat recovery from exhaust is closely related to the flow of exhaust and air. Since these parameters are affected by atmospheric conditions, the efficiency was decreased despite an increase in the exhaust temperature. It can be claimed, however, that the efficiency of heat recovery usually increases along with the temperature of exhaust. The obtained results confirmed that even in the least favourable system (i.e., without the insulation of the flue duct with mineral wool) the chimney works normally. Thanks to the fact that the structure of the analysed chimney allowed the resignation from additional layers of flue insulation and minimised the loss of the heat permeating through the suggested perlite concrete block, it displayed a relatively high efficiency index. As a comparison, the efficiency of the chimney made of leca concrete blocks and insulated with mineral wool which was analysed by Drozdzol [13] was 5.5% and 4.5% for exhaust temperatures of 200 °C and 300 °C, respectively.

### 5.2. Fire Safety

The maximum temperature values recorded on the casing of the chimney and on the wooden elements located at distance x = 50 mm from the chimney are presented in Table 4.

Based on the analysis of fire safety, it may be concluded that the tested chimney made from perlite concrete blocks meets significantly higher requirements than standard EN 13063-1:2005+A1:2007 [40]. During the tests simulating conditions of chimney use, the highest recorded temperature values which were registered 60 min into testing were 68 °C on the block’s surface, and 13 °C on wooden elements located 50 mm from the chimney. Due to the fact that the obtained results were very favourable from the point of view of fire safety, it was decided that the experiment should be continued, and the temperature of the introduced gases was maintained for the next 150 min. At this point, it needs to be noted that the testing conditions were significantly stricter in comparison to the 60 min required by the standard applied during the chimney certification process. One must point out the imperfection of the standard guidelines, as in reality there is a high probability that a heating appliance will operate at high parameters for over 60 min. In spite of the fact that the chimney was subjected to high temperatures for 210 min, the obtained temperature values of 82 °C on the block and 26 °C on the wooden elements located 5 cm from the chimney still met the requirements of standard EN 13063-1:2005+A1:2007 [40]. This confirms the high level of fire safety of the innovative chimney casing.

The next stage of the tests was a 30-min simulation of soot fire, during which exhaust at a temperature exceeding 1000 °C was fed into the chimney. Again, the limit values of the temperature on the surface of the block and on the wooden elements located 5 cm from the chimney were not exceeded, reaching 67 °C on the block and 29 °C on the wooden structure, respectively. In order to provide even more restrictive conditions, the thermocouple on the block was insulated with mineral wool (which limits cooling due to ambient air–stricter conditions compared to standard conditions). The maximum temperature recorded at that point during fire testing was 86 °C. This confirms the high insulation level, and at the same time the fire safety of the designed chimney even in case of failure due to soot fire in the chimney.

The obtained results confirm a higher fire safety level of perlite concrete blocks compared to leca concrete casings commonly used in chimneys. Despite an additional insulation in the form of mineral wool, after 30 min of testing, the temperature of the casings of the chimneys made of leca concrete reached 70 °C, and the temperature of the wooden structure reached 36 °C [13].

### 5.3. Compressive Strength

**Figure 11:**
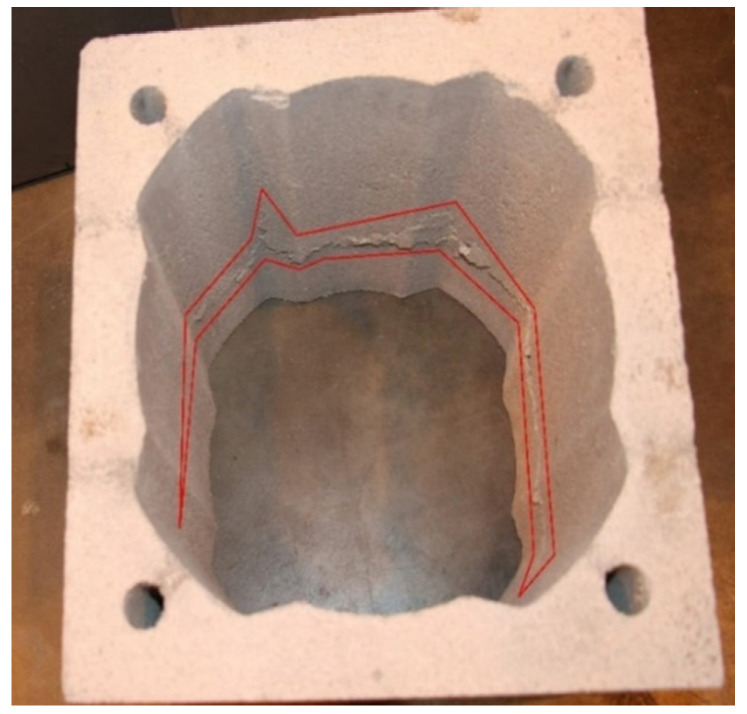
Visible damage caused by compressive load.

**Figure 12:**
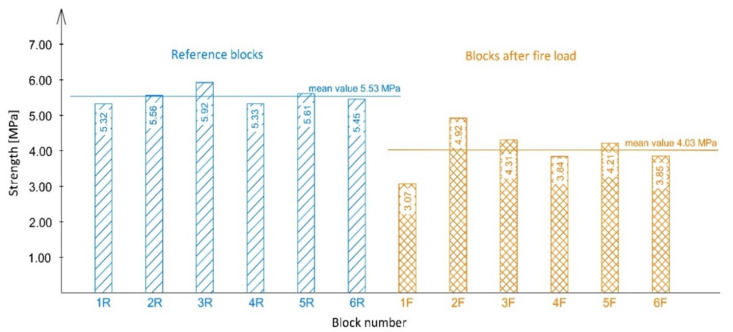
Results of compressive strength tests.

The damage to the block after applying test load is shown in Figure 11. Figure 12 presents the obtained compressive strength values.

The defects created when applying vertical load were observed along the entire internal circumference of each block (Figure 11). The shape of the damage resembled a straight, parallel line. This type of damage means that the tests were carried out correctly and that the samples had been prepared properly. Moreover, it indicates that the load was uniformly applied across the entire surface of the tested sample.

For blocks marked as R (reference), the arithmetic average of compressive strength was 5.53 MPa, and the variability index was determined to be 4%. As for the blocks subjected to fire load (1F–6F), the arithmetic average of compressive strength was 4.03 MPa, and the variability index was determined to be 15%. Both trials were characterised by a variability index lower than 25%, which means that their variability was low.

According to standard EN 12446:2003 [37], the tests are done on cut out corners, however the authors of this study were interested in the true picture of strength, which is why the compressive strength test was done on whole blocks. It was assumed that the chimney’s compressive strength would be lower during fire trials. The strength of perlite concrete blocks which had been subjected to high temperatures was 27% lower on average. The strength of the strongest block which had not been subjected to thermal load was 48% higher than that of the weakest block that had been subjected to fire trial. The lowest compressive strength obtained during testing was 3.07 MPa. Despite a relatively substantial decrease in strength, this value is sufficient for a chimney structure. A block with a strength of 3.07 MPa is capable of bearing a load of a 300-metre-high chimney column. According to standard EN 12446:2003 [37], a chimney should be able to bear a load equal to four times its mass in order to pass the certification process. These types of structures are usually designed to be up to 25 m high and therefore should be able to withstand a load equal to that of their 100 m high counterparts. This means that the chimney strength obtained during testing was at least 3 times higher than that of standard chimneys of the same type, even though the blocks had been weakened due to thermal stresses. It is also worth mentioning that the average compressive strength (4.03 MPa) after fire trial was higher than that of leca concrete blocks readily available on the market, whose strength is approx. 3 MPa at a bulk density of 1200 kg/m^3^ [22,23].

## 6. Conclusions

The results of the experimental tests of efficiency, fire safety and compressive strength allowed the following conclusions:

1. Perlite concrete is an innovative material which has rarely been used in chimney technology. Its application increases the level of insulation and fire safety in comparison with other concretes that have been widely used in chimney technology (e.g., leca concrete).

2. It is possible to recover more heat from perlite concrete chimneys with an air space than from popular leca concrete chimneys. The recovery efficiency determined in the course of our experimental tests was at least 23%. In the case of a leca concrete chimney, this efficiency is determined at 5.5%.

3. The use of perlite concrete in the production of a chimney casing and the use of an air space as insulation ensures fire safety even without additional layers of insulation (e.g., mineral wool). Maximum temperature:

- during fire safety tests in extended operating conditions (exposure to 700 °C with declared temperature of T600) for the duration of 210 min reached 82 °C on the block, and did not exceed 26 °C on the wooden structure located 50 mm away from the chimney,
- during soot fire simulation (introduction of exhaust at over 1000 °C to the flue) reached 67 °C on the block, and 29 °C on the wooden structure located 50 mm away from the chimney.

4. Although the perlite concrete block was weakened due to exposure to very high temperatures that can be present in a chimney during a soot fire, it still displayed (over 3 times) higher compressive strength parameters than it is required by applicable standards. Moreover, the lowest value of its compressive strength (3.07 MPa) after subjecting the block to high temperatures is comparable to the average compressive strength declared by the manufacturers of widely used leca concrete blocks (3 MPa). It is worth emphasising that the average compressive strength of the analysed block was approx. 1 MPa higher than that of blocks readily available on the market.

5. The lower mass of perlite concrete blocks has a considerable effect on the cost of transport to the construction site. The costs of chimneys with perlite concrete casings are further lowered by the ability to resign from mineral wool. Additionally, in case of application of the air layer in the chimney, resignation from wool insulation increases the chimney’s fire safety. Furthermore, lighter blocks facilitate chimney construction, and the structure of perlite concrete blocks allows easier and more aesthetically pleasing machining compared to leca concrete blocks.

## Figures and Tables

**Table 1:** Physical characteristics of perlite used in the production of blocks.

Colour	White
Refractive index	1.5
pH	6.5–8.0
Moisture	0.5%
Specific gravity:	2.2–2.4 kg/m^3^
Bulk density of crude ore	960–1200 kg/m^3^
Bulk density of expanded perlite	60–160 kg/m^3^
Particle size	<2.5 mm
Softening point	871–1093 °C
Fusion point	1260–1343 °C
Specific heat	837 J/kg·K
Thermal conductivity (λ)	0.045–0.065 W/m·K
Bulk density	90–110 kg/m^3^
Grain size	Amount in volume
+2 mm	Min. 25%
+1 mm	Min. 85%
+0.5 mm	Min. 90%
-0.5 mm	Max 10%

**Table 2:** Main characteristics of the analysed perlite and leca concrete samples.

Sample Number	Bulk Density (kg/m^3^)	Δ*T*_1_ (°K)	Δ*T*_2_ (°K)	Average Temperature in the Sample (°K)	Thermal Conductivity Coefficient (W/m·K)	Compressive Strength (MPa)
Perlite in Loose State						
1	144	9.4	10.8	301.25	0.057	-
leca concrete blocks						
2	1042	6.4	9.9	300.45	0.45	3.00–3.50 [34,35]
Perlite Concrete Blocks						
3	943	7.4	10.5	299.85	0.22	1.86
4	1039	6.4	9.7	300.35	0.37	3.68
5	1144	6.6	10.2	299.65	0.39	3.92
6	1294	5.2	8.6	300.55	0.67	4.44

**Table 3:** Temperature values recorded during tests of the chimney’s thermal efficiency.

Time		Exhaust Temperature at Outlet (°C)	Air Temperature at Inlet (°C)
Hour	Minute		
1 h	30	160	51
	45	161	52
	60	168	53
2 h	75	170	54
	90	171	54
	105	173	55
3 h	120	215	63
	135	238	64
	150	243	70
4 h	165	268	84
	180	272	86
	195	284	94

**Table 4:** Results of fire tests in operating conditions T600 (700 °C) and in soot fire conditions (1000 °C).

Time	Temperature at Inlet (°C)	Maximum Temperature Recorded on the Surface of: (°C)	
		Blocks	Wooden Elements
**Operating Conditions T600 (700 °C)**			
30’	706	62	13
60’	708	68	16
90’	708	76	22
120’	709	77	23
150’	708	79	25
190’	708	81	25
210’	709	82	26
**Soot fire at 1000 °C**			
10’	1002	46	17
20’	1005	48	27
30’	1001	67	29

## Data Availability

Data is contained within the article.
